# Mental Health, Socioeconomic Position, and Oral Health: A Path Analysis

**DOI:** 10.5888/pcd21.240097

**Published:** 2024-10-03

**Authors:** Lisa J. Heaton, Morgan Santoro, Tamanna Tiwari, Rebecca Preston, Kelly Schroeder, Cameron L. Randall, Adrianna Sonnek, Eric P. Tranby

**Affiliations:** 1 CareQuest Institute for Oral Health, Boston, Massachusetts; 2 School of Dental Medicine, University of Colorado Anschutz Medical Campus, Aurora; 3 University of Washington School of Dentistry, Seattle

## Abstract

**Introduction:**

Mental health conditions and poor oral health outcomes share bidirectional links, and both are linked to factors related to socioeconomic position (SEP). We used nationally representative survey data to describe the complex interplay of SEP, mental health, oral health behaviors, dental treatment seeking, and oral health.

**Methods:**

We used data from the 2022 State of Oral Health Equity in America survey, which collects data from US adults on prior depression diagnosis and current depressive symptoms via the Patient Health Questionnaire-9 and demographic characteristics (age, sex/gender, race, ethnicity), SEP (education, income, employment, home ownership, dental insurance), oral health behaviors (brushing and flossing frequency), dental treatment seeking (time since last visit, plans for visit in the coming year), and self-rated oral health (feeling self-conscious due to poor oral health, having symptoms of poor oral health). We used structural equation modeling to identify latent variables and fit the path analytic models.

**Results:**

In the total sample (N = 5,682), SEP was significantly associated with dental treatment seeking (standardized parameter estimate [SE] = 0.55 [0.05]), oral health behaviors (standardized parameter estimate [SE] = 0.34 [0.04]), and mental health (standardized parameter estimate [SE] = 0.59 [0.05]). These factors, in turn, were significantly associated with self-rated oral health (estimates ranging from 0.20 to 0.54, SEs ranging from 0.04 to 0.05).

**Conclusion:**

SEP, which involves several major social determinants of health, is directly associated with mental health and indirectly associated with self-rated oral health status, with mental health modifying the relationship between SEP and self-rated oral health. Findings emphasize the need to integrate medical, dental, and behavioral health with the goal of providing comprehensive person-centered care.

**Summary**

**What is already known on this topic?**

Mental health conditions and poor oral health outcomes share bidirectional links, and both are linked to factors related to socioeconomic position (SEP).

**What is added by this report?**

We used nationally representative survey data and path analysis to examine the complex interplay of SEP, mental health, oral health behaviors, dental treatment seeking, and oral health. We demonstrated that SEP is significantly associated with dental treatment seeking, oral health behaviors, and mental health. These factors, in turn, are significantly associated with self-rated oral health.

**What are the implications for public health practice?**

Our findings emphasize the need to integrate medical, dental, and behavioral health to provide comprehensive person-centered care.

## Introduction

Depressive disorders, such as major depressive disorder (MDD) and persistent depressive disorder (dysthymia), negatively affect a person’s functioning and are characterized by “sad, empty, or irritable mood” as well as physical and cognitive changes (1). A diagnosis of MDD — the most prevalent type of depressive disorder — requires experiencing symptoms for 2 weeks or more, including depressed mood, loss of interest in usual activities, too much or too little sleep, changes in appetite, lack of energy or concentration, and feelings of worthlessness, among others (1).

The National Institutes of Health estimates that in 2021, the most recent year for which data are available, 8.3% of US adults aged 18 or older (approximately 21 million adults) experienced at least 1 MDD episode (2). Females are more likely than males to be diagnosed with MDD (10.3% vs 6.2% in 2021), and adults aged 18 to 25 years are more likely than adults aged 26 years or older to be diagnosed (2).

MDD and depressive symptoms are strongly associated with poor oral health outcomes, including increased dental caries (decay) (3), periodontal (gum) disease (4), and missing teeth due to dental disease (5). The association between depression and poor oral health outcomes is, as with most associations, multifactorial. Individuals with depression are less likely than individuals without depression to seek regular dental care (6), even when they perceive they need dental treatment (7). Depression is associated with less frequent oral health behaviors such as toothbrushing and flossing (8), likely due to a lack of motivation, which is a hallmark of MDD (1). Many medications used to treat MDD lead to xerostomia (dry mouth), which is linked to an increased risk of dental caries (9). Perhaps unsurprising, then, individuals with MDD are more likely than individuals without MDD to rate their oral health as poor (10).

In addition to poorer self-rated oral health, depression is strongly linked with poor oral health–related quality of life (OHRQoL), which has been defined as “comfort when eating, sleeping and engaging in social interaction; their self-esteem; and their satisfaction with respect to their oral health” (11). Depression is significantly associated with lower OHRQoL in both females and males (12). Depression is linked with feeling embarrassed when showing teeth while smiling and laughing, as well as having difficulty at work or school due to oral health problems (13). Furthermore, adults missing at least 1 permanent tooth, a standard item on most measures of OHRQoL, are significantly more likely than adults with a complete dentition to report depressive symptoms (6).

Depression and poor oral health outcomes are both linked to factors related to socioeconomic position (SEP). For example, lower income is associated with both elevated depressive symptoms (as measured by assessments such as the 9-item Patient Health Questionnaire [PHQ-9]) (14) and a higher prevalence of caries (15). Home ownership, a proxy for “secure and sustainable housing,” is associated with lower levels of depression and better quality of life compared with renting one’s home (16). Similarly, individuals who own rather than rent their home are more likely to have visited a dentist within the previous year (17). Unemployment is associated with irregular dental attendance and greater numbers of missing and decayed teeth (18). Higher levels of education (ie, at least some college) appear to protect against depressive symptoms, particularly among individuals from disadvantaged backgrounds (19). Meanwhile, individuals with lower levels of education have a higher risk of periodontal disease than those with higher education (20). Research is lacking on the link between depression and having dental insurance, in part because dental insurance status is most often controlled for as a confounder in studies of depression and oral health. However, individuals without dental insurance are less likely than those with dental insurance to have visited a dentist in the previous year but more likely to visit a dentist for pain relief (21).

Given the important and complex relationship between depression and oral health, it is essential to understand the interplay of factors such as SEP, dental treatment seeking, oral health behaviors, and oral health as they relate to depressive symptoms and perceived oral health. The scientific literature addresses individual associations between these variables, but it does not address the complexities and potential pathways. Thus, the objective of this study was to examine the interrelationships between these factors in a nationally representative survey of US adults.

## Methods

### State of Oral Health Equity in America survey

We conducted a nationally representative survey in January and February 2022. The State of Oral Health Equity in America (SOHEA) survey contains approximately 150 questions and collects data on consumers’ experiences, attitudes, and behaviors related to oral health (22). An internal team from CareQuest Institute for Oral Health developed the survey questions, and the questions were pretested internally at CareQuest Institute and pilot tested by the research organization National Opinion Research Center (NORC) at the University of Chicago on an initial sample (N = 48). This study protocol was reviewed and determined to be exempt from human subjects review by the WCG Institutional Review Board.

The SOHEA survey was administered online (96.7%) and via telephone (3.3%) by NORC through their nationally representative AmeriSpeak panel (23). Participants were consumers aged 18 years or older who were part of the probability-based panel designed to be representative of the US household population. Area probability and address-based sampling were used by NORC to randomly select US households from within its known, nonzero probability of selection from the NORC National Sample Frame. Therefore, respondents were enrolled as AmeriSpeak panelists by NORC separately from and before the current study according to NORC’s recruitment guidelines (23). For the current study, NORC used an initial sampling unit of 17,603 and invited 1 adult from each eligible household in the AmeriSpeak panel to participate in the survey, either online or by telephone. At the time of initial recruitment, AmeriSpeak panelists indicate whether they would like to participate in surveys online or via telephone; they were invited to participate in the current study according to these preferences. Those preferring to take surveys online were sent a link to the survey, and those who preferred to complete the survey via telephone were contacted by NORC staff members (trained in survey administration by NORC) by telephone. The margin of error for the survey was 1.75%, and the survey completion rate was 32.3%. Respondents were offered $5 for their participation.

### Measures

Demographic variables included age in years (categorized as 18–29, 30–44, 45–59, or ≥60 y); gender (male, female, transgender, or do not identify as male, female, or transgender); and race and ethnicity (non-Hispanic Asian, non-Hispanic Black, Hispanic, non-Hispanic White, non-Hispanic Other, or ≥2 races non-Hispanic). 

The mental health factor comprised 3 variables: 1) scores on the PHQ-9 (24), 2) responses to a question about self-rated mental or emotional health (categorized as excellent, very good, good, fair, or poor), and 3) whether the respondent had ever been diagnosed with depression (yes or no). The PHQ-9 is a 9-item scale that asks respondents to indicate how frequently in the previous 2 weeks (not at all, several days, more than half the days, or nearly every day) they had experienced each of the DSM-5 (*Diagnostic and Statistical Manual of Mental Disorders, Fifth Edition* [25]) criteria for MDD. The PHQ-9 has good internal reliability and construct validity (24).

The SEP factor comprised 5 variables: 1) annual household income (<$30,000, $30,000 to <$60,000, $60,000 to <$100,000, or ≥$100,000); 2) home ownership (own home, rent home, or occupied current home without payment of cash rent); 3) employment status (working as a paid employee or self-employed, not working due to layoff or looking for work, not working due to being retired, disabled, or other); 4) education (less than high school, high school graduate or equivalent, vocational/technical school/some college/associate degree, bachelor’s degree, or postgraduate study/professional degree); and 5) dental insurance (yes or no).

The dental treatment seeking factor comprised 2 variables: 1) time since most recent dental visit (within the past year or >1 year ago) and 2) whether the respondent plans to visit a dentist in the coming year for routine or preventive care (yes or no). The oral health behavior factor comprised 2 variables: frequency of toothbrushing (twice per day or more, once per day or less) and frequency of flossing (once per day or more, less than once a day).

The OHRQoL factor was measured by assessing answers to 2 questions. The first one asked, “How often in the last year have you been self-conscious or embarrassed because of your teeth, mouth, or dentures?” Response options were very often, fairly often, occasionally, hardly ever, or never. The second question asked about the number of missing permanent teeth due to tooth decay or gum disease; response options were none or 1 or more.

Respondents indicated their perceived (self-rated) oral health by answering the question, “In general, how would you rate your oral health (state of your teeth, mouth and gums)?” Response options included excellent, very good, good, fair, or poor. Having at least 1 symptom of poor oral health was measured with the following question: “In the last twelve months, have you ever had any of the following symptoms?” Options (yes or no) included swollen or bleeding gums; pain when chewing or swallowing; chronic bad breath; toothache; cracked or broken teeth; swelling of the face or cheek; clicking of the jaw or temporomandibular joint pain; frequent dry mouth; or ulcers, sores, or tender areas in the mouth that do not heal on their own. Respondents were noted to have at least 1 oral health problem if they answered yes to 1 or more of these oral health problems.

### Statistical analyses

We used descriptive statistics to summarize the percentage and frequencies for demographic variables by PHQ-9 scores dichotomized into 0 to 9 (“minimal”) and 10 to 27 (“elevated”). We used this dichotomy because a score of 10 to 27 suggests a possible MDD diagnosis (24). We conducted χ^2^ analyses to test differences in variables related to mental health, dental treatment seeking, oral health behaviors, OHRQoL, and self-rated oral health by dichotomized PHQ-9 scores. 

**Figure 1:**
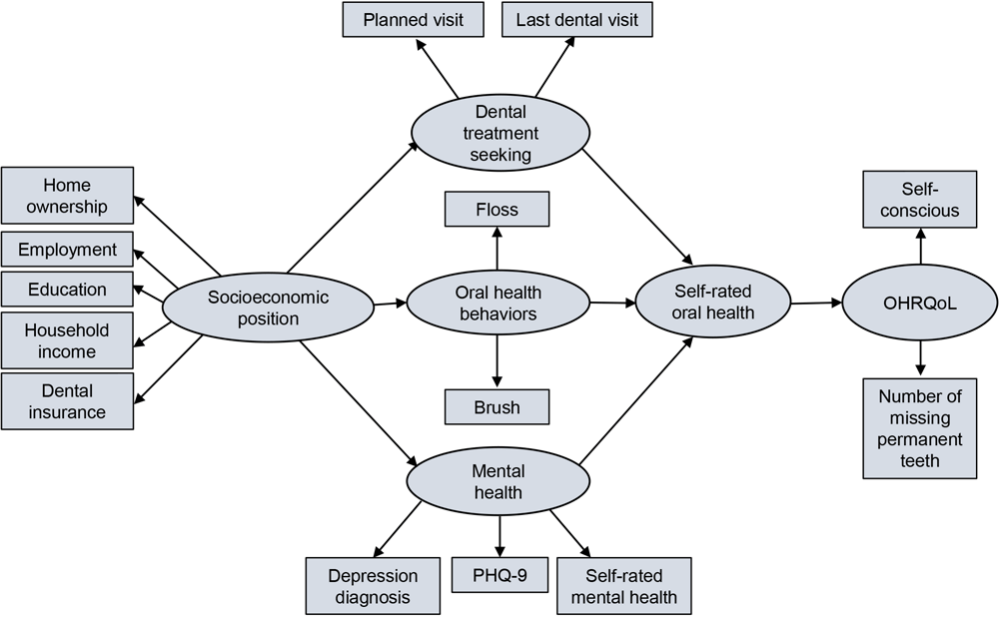
Theoretical model of proposed pathways linking socioeconomic position, dental treatment seeking, oral health behaviors, mental health, and self-rated oral health with OHRQoL. Abbreviations: PHQ-9, Patient Health Questionaire-9; OHRQoL, oral health–related quality of life.

We used structural equation modeling (SEM) to test our theoretical model. Because of the categorical variables in our model, we used the asymptotic distribution-free method. We developed the theoretical model (Figure 1) for our study on the basis of a review of the literature, with input from dentists, clinical psychologists, sociologists, and data scientists with expertise in mental health and dental public health. All missing data were set to null and so were not captured in the analysis.

Stage 1 involved using confirmatory factor analytic models to separately specify latent variables and outcomes. In this step, we assessed all standardized factor loadings. In stage 2, we fit the path analytic models, including the latent constructs, to jointly estimate the direct and indirect associations of the latent variables on the outcome variable. To compare their relative sizes, we derived weighted standardized model parameters. We used the comparative fit index (CFI), root mean square error of approximation (RMSEA), and standardized root mean square residual (SRMR) to assess goodness of fit, with CFI greater than 0.95 and RMSEA and SRMR indicating good model fit. We conducted all analyses by using RStudio 4.2.0 (R Core Team, R Foundation for Statistical Computing).

## Results

The study sample consisted of 5,682 adults. Just more than half (50.3%) of the survey respondents identified as female, and the age distribution had bimodal peaks at 30 to 44 years (30.8%) and 60 years or older (31.4%) (Table 1). More than half (59.5%) of the sample identified as non-Hispanic White, followed by those identifying as Hispanic (16.2%), non-Hispanic Black (10.4%), 2 or more races (5.7%), Other race and ethnicity (5.2%), and non-Hispanic Asian (3.0%). Most respondents had some college or more (77.7%), and household income was evenly distributed across 4 levels. Most respondents were employed (59.5%), and nearly two-thirds owned their own homes (63.2%). Finally, most respondents reported having dental insurance (70.5%).

**Table 1:** Demographic and SEP Characteristics of Sample (N = 5,682), by Patient Health Questionnaire–9 Scores, State of Oral Health Equity in America Survey, January–February 2022

Characteristic	No. (%)^a^		
	Total	Minimal score (0-9)^b^	Elevated score (10–27)^b^
**Demographic**			
**All**	5,682	4,568 (81.9)	1,009 (18.1)
**Age group, y**			
18–29	934 (16.7)	635 (13.9)	299 (29.0)
30–44	1,722 (30.8)	1,317 (28.9)	405 (39.3)
45–59	1,178 (21.1)	994 (21.8)	184 (17.9)
≥60	1,752 (31.4)	1,610 (35.3)	142 (13.8)
**Sex/gender**			
Male	2,725 (48.8)	2,299 (50.5)	426 (41.4)
Female	2,810 (50.3)	2,230 (49.0)	580 (56.3)
Transgender	18 (0.3)	12 (0.3)	6 (0.6)
Do not identify as male, female, or transgender	29 (0.5)	11 (0.2)	18 (1.7)
**Race and ethnicity**			
Hispanic	906 (16.2)	695 (15.3)	211 (20.5)
Non-Hispanic Asian	168 (3.0)	135 (3.0)	33 (3.2)
Non-Hispanic Black	581 (10.4)	471 (10.3)	110 (10.7)
Non-Hispanic White	3,322 (59.5)	2,813 (61.7)	509 (49.4)
Non-Hispanic Other	288 (5.2)	207 (4.5)	81 (7.9)
≥2 Races, non-Hispanic	321 (5.7)	235 (5.2)	86 (8.3)
**SEP**			
**Education**			
Less than high school	297 (5.3)	206 (4.5)	91 (8.8)
High school graduate or equivalent	950 (17.0)	705 (15.5)	245 (23.8)
Some college or associate degree	2,368 (42.4)	1,898 (41.7)	470 (45.6)
Bachelor’s degree	1,183 (21.2)	1,035 (22.7)	148 (14.4)
Postgraduate/professional degree	788 (14.1)	712 (15.6)	76 (7.4)
**Annual household income, $**			
<30,000	1,370 (24.5)	960 (21.1)	410 (39.8)
30,000 to <60,000	1,537 (27.5)	1,241 (27.2)	296 (28.7)
60,000 to <100,000	1,411 (25.3)	1,206 (26.5)	205 (19.9)
≥100,000	1,268 (22.7)	1,149 (25.2)	119 (11.6)
**Employment status**			
Working/paid employee or self-employed	3,324 (59.5)	2,759 (60.6)	565 (54.9)
Not working/laid off or looking for work	406 (7.3)	271 (5.9)	135 (13.1)
Not working/retired/disabled/other	1,856 (33.2)	1,526 (33.5)	330 (32.0)
**Home ownership**			
Own home	3,530 (63.2)	3,075 (67.5)	455 (44.2)
Rent	1,870 (33.5)	1,351 (29.7)	519 (50.4)
Occupy without payment of rent	186 (3.3)	130 (2.9)	56 (5.4)
**Dental insurance**			
Yes	3,922 (70.5)	3,266 (72.0)	656 (64.2)
No	1,638 (29.5)	1,272 (28.0)	366 (35.8)

Abbreviation: SEP, socioeconomic position.

a Not all respondents answered all questions. Categories may not add to totals; percentages are based on number of respondents who answered questions; percentages may not add to 100 because of rounding.

b All differences between minimal score and elevated score were significant at *P* < .001; determined by χ^2^ test.

All factors of interest (mental health, dental treatment seeking, oral health behaviors, OHRQoL, and self-rated oral health) were significantly associated with PHQ-9 scores (all *P* values <.001; Table 2). Respondents with self-rated excellent/very good/good mental health had minimal PHQ-9 scores in larger proportions (89.0%) than respondents with elevated PHQ-9 scores (50.4%), and respondents who reported having ever been diagnosed with depression had elevated PHQ-9 scores in larger proportions (91.7%) than respondents without a depression diagnosis (78.7%).

**Table 2:** Mental Health and Oral Health Characteristics of the Sample (N = 5,682), by Patient Health Questionnaire–9 Scores, State of Oral Health Equity in America Survey, January–February 2022

Characteristic	Total, no. (%)^a^	No. (%) [95% CI]^b^	
		Minimal score (0–9)	Elevated score (10–27)
**Mental health**			
**Self-rated mental health**			
Excellent/very good/good	4,564 (82.0)	4,056 (89.0) [87.7–90.1]	508 (50.4) [46.2–54.7]
Fair/poor	1,002 (18.0)	502 (11.0) [9.9–12.3]	500 (49.6) [45.3–53.8]
**Ever diagnosed with depression**			
Yes	581 (84.7)	292 (78.7) [72.6–83.6]	289 (91.7) [87.5–94.6]
No	105 (15.3)	79 (21.3) [16.4–27.4]	26 (8.3) [5.4–12.5]
**Dental treatment seeking**			
**Last dental visit**			
≤1 Year ago	3,624 (65.0)	3,113 (68.2) [66.4–70.0]	511 (50.6) [46.4–54.9]
>1 Year ago	1,948 (35.0)	1,450 (31.8) [30.0–33.6]	498 (49.4) [45.1–53.6]
**Plan to see dentist in coming year**			
Yes	4,719 (94.3)	3,951 (95.4) [94.5–96.1]	768 (88.9) [85.2–91.7]
No	287 (5.7)	191 (4.6) [3.9–5.2]	96 (11.1) [8.3–14.8]
**Oral health behaviors**			
**Toothbrushing**			
≥2 Times per day	3,569 (64.2)	3,015 (66.2) [64.4–68.0]	554 (54.9) [50.7–59.1]
≤1 Time per day	1,992 (35.8)	1,537 (33.8) [32.0–35.6]	455 (45.1) [40.9–49.3]
**Flossing**			
≥1 Time per day	2,468 (44.4)	2,079 (45.6) [43.7–47.6]	389 (38.6) [34.6–42.9]
<1 Time per day	3,096 (55.6)	2,478 (54.4) [52.4–56.3]	618 (61.4) [61.3–65.4]
**Oral health-related quality of life**			
**Felt self-conscious**			
Very often	419 (7.6)	230 (5.1) [4.2–6.1]	189 (19.1) [15.9–22.8]
Fairly often	383 (6.9)	235 (5.2) [4.4–6.1]	148 (15.0) [12.1–18.4]
Occasionally	1,020 (18.5)	793 (17.5) [16.1–19.1]	227 (23.0) [19.7–26.6]
Hardly ever	1,428 (25.9)	1,224 (27.1) [25.4–28.8]	204 (20.6) [17.5–24.2]
Never	2,263 (41.0)	2,043 (45.1) [43.2–47.1]	220 (22.3) [18.7–26.2]
**No. of missing permanent teeth**			
None	3,008 (54.1)	2,520 (55.4) [53.4–57.3]	488 (48.4) [44.2–52.6]
≥1	2,550 (45.9)	2,030 (44.6) [42.7–46.6]	521 (51.6) [47.4–55.8]
**Self-rated oral health**			
Excellent/very good/good	4,170 (74.8)	3,602 (78.9) [77.3–80.5]	568 (56.3) [52.1–60.5]
Fair/poor	1,402 (25.2)	962 (21.1) [19.5–22.7]	440 (43.7) [39.5–47.9]

a Not all respondents answered all questions. Categories may not add to totals; percentages are based on number of respondents who answered questions; percentages may not add to 100 because of rounding.

b All differences between minimal score and elevated score were significant at *P* < .001; determined by χ^2^ test.

Respondents with minimal PHQ-9 scores were more likely to report having a dental visit within the previous year (68.2%) and more likely to report planning to visit a dentist in the coming year (95.4%) compared with those with elevated PHQ-9 scores (50.6% and 88.9%, respectively). Respondents with minimal PHQ-9 scores were more likely to report brushing their teeth twice per day or more (66.2%) and flossing their teeth once per day or more (45.6%) than respondents with elevated PHQ-9 scores (54.9% and 38.6%, respectively).

Respondents with elevated PHQ-9 scores were more likely to report they very often felt self-conscious or embarrassed because of their teeth, mouth, or dentures (19.1%) compared with respondents with minimal PHQ-9 scores (5.1%). Similarly, those with elevated PHQ-9 scores were more likely than those with minimal PHQ-9 scores to have 1 or more missing permanent teeth (51.6% and 44.6%, respectively). Respondents with elevated PHQ-9 scores were also more likely to describe their oral health as fair/poor (43.7%) compared with those with minimal PHQ-9 scores (21.1%).

The initial model (Figure 1) did not converge when initially assessed, indicating it was not the best-fitting model. After using SEM to test the theoretical model, we determined that OHRQoL and one of its corresponding variables (the number of missing permanent teeth) did not fit within the model.

**Figure 2:**
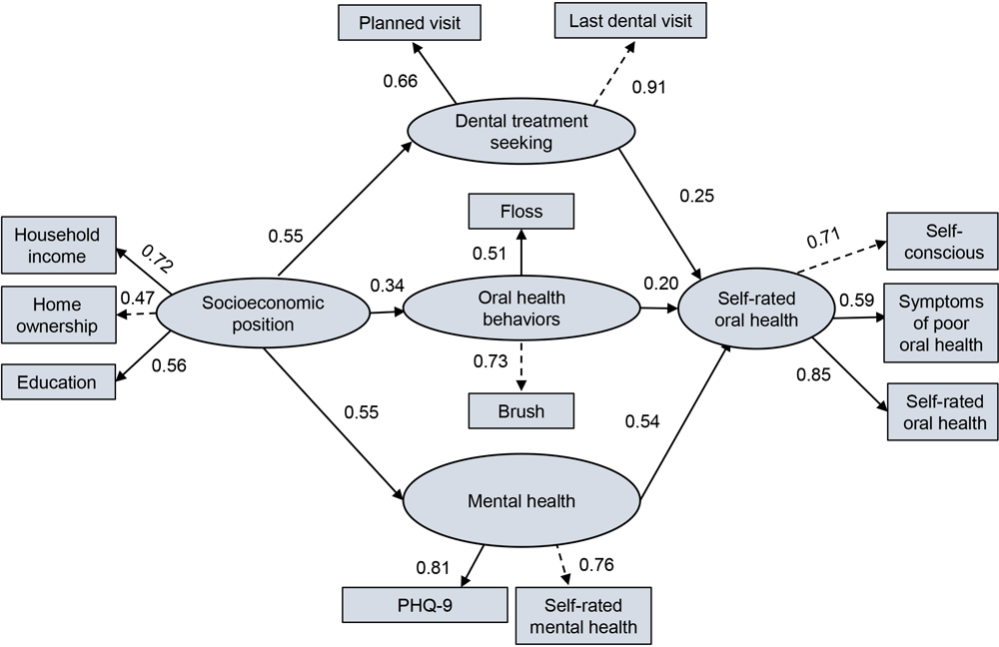
Final model with standardized parameter estimates of pathways linking socioeconomic position (SEP), dental treatment seeking, oral health behaviors, and mental health with self-rated oral health. Dotted lines indicate fixed parameter factor loading. Solid lines indicate predictive relationships between variables and that changes in the predictor variable are associated with changes in the outcome variable. Abbreviations: PHQ-9, Patient Health Questionaire-9; OHRQoL, oral health–related quality of life.

Stage 1 of SEM then involved using confirmatory factor analytic models to separately specify the 4 latent variables (SEP, dental treatment seeking, oral health behaviors, mental health) and revised outcome (self-rated oral health status, measured by self-rated oral health, having at least 1 symptom of poor oral health, and feeling self-conscious due to poor oral health). In this step, we assessed all standardized factor loadings. In Stage 2, we fit the path analytic models, including the latent constructs, to jointly estimate the direct and indirect associations of 4 latent variables on self-rated oral health status (Figure 2). The final model had a good fit, with a CFI of 0.96, an RMSEA of 0.04, and an SRMR of 0.07.

SEP was associated with dental treatment seeking (standardized parameter estimate [SE] = 0.55 [0.05]), oral health behaviors (standardized parameter estimate [SE]= 0.34 [0.04]), and mental health (standardized parameter estimate [SE] = 0.55 [0.05]) (Table 3). These factors, in turn, were associated with self-rated oral health (standardized parameter estimates ranging from 0.20 to 0.54, SEs ranging from 0.04 to 0.05).

**Table 3:** Standardized Parameter Estimates of Pathways Linking SEP, Dental Treatment Seeking, Oral Health Behaviors, and Mental Health With Self-Rated Oral Health: Results of Structural Equation Model^a^^,^ b

Pathway^c^	Parameter estimate (SE)	Standardized parameter estimate (SE)
SEP → Household income	1	0.72 (0.02)
SEP → Home ownership	0.65 (0.04)	0.47 (0.02)
SEP → Education	0.77 (0.04)	0.56 (0.02)
Dental treatment seeking → Last dental visit	1	0.91 (0.06)
Dental treatment seeking → Planned dental visit	0.73 (0.08)	0.66 (0.05)
Oral health behaviors → Brush	1	0.73 (0.06)
Oral health behaviors → Floss	0.70 (0.11)	0.51 (0.04)
Mental health →Self-rated mental health	1	0.76 (0.03)
Mental health → PHQ-9	1.06 (0.06)	0.81 (0.03)
Self-rated oral health → Self-conscious	1	0.71 (0.02)
Self-rated oral health → Self-rated oral health	1.20 (0.05)	0.85 (0.02)
Self-rated oral health → Symptoms of poor oral health	0.82 (0.05)	0.59 (0.03)
SEP → Dental treatment seeking	0.70 (0.05)	0.55 (0.04)
SEP → Oral health behaviors	0.34 (0.04)	0.34 (0.04)
SEP → Mental health	0.59 (0.05)	0.55 (0.03)
Self-rated oral health ← Dental treatment seeking	0.20 (0.04)	0.25 (0.04)
Self-rated oral health status ← Oral health behaviors	0.20 (0.04)	0.20 (0.04)
Self-rated oral health ← Mental health	0.50 (0.05)	0.54 (0.04)

Abbreviation: SEP, socioeconomic position.

a Comparative fit index = 0.96; root mean square of approximation = 0.04; standardized root mean square residual = 0.07.

b Data were from a sample of respondents (N = 5,682) to the State of Oral Health Equity in America Survey, January–February 2022.

c Arrows indicate direction of predictive relationship between variables.

## Discussion

This study evaluated the relationships among SEP, dental treatment seeking, oral health behaviors, and mental health, and how these factors are associated with self-rated oral health status. This study is the first to use path analysis and data from a nationally representative survey to understand these complex interrelationships and their effect on oral health.

Among adults who completed the SOHEA survey, 18.0% rated their mental health as fair or poor. These results are generally consistent with data from the Centers for Disease Control and Prevention and the National Institute of Mental Health, which reported that approximately 1 of 5 US adults live with a mental illness (26). In our study, 10.2% (581 of 5,682) of all study respondents reported they had ever been diagnosed with depression, somewhat lower than the 18.4% of 2020 Behavioral Risk Factor Surveillance System participants reporting a depression diagnosis at any point in their lifetime (27). Sampling approaches, question wording, recall bias, or other factors may account for this difference in reports of lifetime depression diagnosis (28,29). Regardless, an elevated PHQ-9 score was significantly associated with other variables in our study in the same direction as found in the existing literature (8,10–12,16).

Interestingly, we found that SEP, which involves several major social determinants of health, was directly associated with mental health and indirectly associated with self-rated oral health status, with mental health modifying the relationship between SEP and self-rated oral health. The finding that mental health and oral health are associated with each another is consistent with evidence from the US and globally (30). Our finding that SEP is linked to both mental and oral health in complex ways extends knowledge of this connection by suggesting that both mental and oral health share common social determinants of health. This finding builds on previous studies of individual associations showing that education, income, and employment are common social determinants of mental and oral health (31).

Oral health behavior — specifically the hygiene-related behaviors of toothbrushing and flossing — modified the relationship between SEP and self-rated oral health, which included oral health symptoms of poor oral health and feelings of self-consciousness due to poor oral health, a component of OHRQoL. OHRQoL is affected by several oral health behaviors, including smoking, alcohol use, and oral hygiene (32), especially toothbrushing (33). A study using structural equation modeling analysis reported a similar pathway between socioeconomic status (SES), frequency of toothbrushing, and quality of life, including self-esteem related to poor oral health. SES also directly predicted a higher frequency of toothbrushing, which was related to better quality of toothbrushing (33). Other research showed similar results relating oral hygiene behaviors to OHRQoL (34).

Also interesting was the finding that dental treatment seeking was directly associated with SEP and modified the relationship between SEP and self-rated oral health. Dental treatment seeking is highly dependent on income, health insurance, type of health insurance, and treatment affordability (35). Seeking dental treatment is also closely related to beliefs, attitudes, and emotions. For example, perception of disease severity, perception of barriers to seeking care, and dental fear and anxiety influence dental treatment seeking (36). Strong evidence from past research shows that lower levels of dental treatment seeking are associated with dental caries and tooth loss, outcomes that are closely associated with OHRQoL (37).

### Limitations

This study has 2 main limitations. First, some factors included in the tested model comprised only 1 or 2 variables, each assessed with just a single survey item. To assess OHRQoL, the survey asked 2 questions developed by the research team instead of using lengthy previously published instruments (eg, the Oral Health Impact Profile [38]). Had we used one of these lengthy instruments to measure variables such as oral health behaviors and OHRQoL, we may have obtained richer data. However, the administration of a lengthy, comprehensive survey had to be balanced with minimizing participant burden. Furthermore, the single-item measures developed for the SOHEA survey were pretested and pilot-tested before administration, ensuring data quality similar to that obtained from such instruments as the PHQ-9. The second main limitation is that this study relied entirely on self-reported data and did not include a clinical assessment of oral health status. Self-reported data can be susceptible to response biases, such as social desirability bias. The telephone administration of this survey may have somewhat mitigated this bias. Moreover, although a clinical assessment of oral health status using a gold-standard tool would be worthwhile for future studies on this topic, self-perception of oral health is an important outcome (10).

### Conclusion

This study described a well-fitting model that demonstrated associations between SEP and mental health, oral health behaviors, and dental treatment seeking, which were, in turn, associated with oral health. These findings further highlight the role of major social determinants of health, such as SEP, for both mental health and oral health. Additionally, these findings advance the field by identifying the pathways and modifiers of the complex relationships among social determinants of health, behavioral determinants of health, mental health, and oral health. This study provides further evidence that oral health is part of overall health and that oral health and mental health are connected in multifaceted ways, underscoring the need to find integrated and person-centered ways to treat them. Research that uses self-reported data and behavioral and clinical assessments could build on this study to further explore the complex associations among social determinants of health, health behaviors, mental health, and oral and systemic health, and identify populations and approaches for intervention.
